# Bestatin, A Pluripotent Immunomodulatory Small Molecule, Drives Robust and Long-Lasting Immune Responses as an Adjuvant in Viral Vaccines

**DOI:** 10.3390/vaccines11111690

**Published:** 2023-11-04

**Authors:** Hyeong Won Kim, Mi-Kyeong Ko, So Hui Park, Seokwon Shin, Su-Mi Kim, Jong-Hyeon Park, Min Ja Lee

**Affiliations:** Center for Foot-and-Mouth Disease Vaccine Research, Animal and Plant Quarantine Agency, 177 Hyeoksin 8-ro, Gimcheon-si 39660, Gyeongsangbuk-do, Republic of Korea; khw6848@korea.kr (H.W.K.); mkk80@korea.kr (M.-K.K.); sohui33@korea.kr (S.H.P.); seokwon1218@korea.kr (S.S.); beliefsk@korea.kr (S.-M.K.); parkjhvet@korea.kr (J.-H.P.)

**Keywords:** bestatin, adjuvant, foot-and-mouth disease, inactivated vaccine, cellular and humoral immunity

## Abstract

An inactivated whole-virus vaccine is currently used to prevent foot-and-mouth disease (FMD). Although this vaccine is effective, it offers short-term immunity that requires regular booster immunizations and has several side effects, including local reactions at the vaccination site. To address these limitations, herein, we evaluated the efficacy of bestatin as a novel small molecule adjuvant for inactivated FMD vaccines. Our findings showed that the FMD vaccine formulated with bestatin enhanced early, intermediate-, and particularly long-term immunity in experimental animals (mice) and target animals (pigs). Furthermore, cytokines (interferon (IFN)α, IFNβ, IFNγ, and interleukin (IL)-29), retinoic acid-inducible gene (RIG)-I, and T-cell and B-cell core receptors (cluster of differentiation (CD)28, CD19, CD21, and CD81) markedly increased in the group that received the FMD vaccine adjuvanted with bestatin in pigs compared with the control. These results indicate the significant potential of bestatin to improve the efficacy of inactivated FMD vaccines in terms of immunomodulatory function for the simultaneous induction of potent cellular and humoral immune response and a long-lasting memory response.

## 1. Introduction

Foot-and-mouth disease (FMD), a viral disease caused by the FMD virus (FMDV), is characterized by high transmissibility and a wide host range. More than 70 cloven hoof animals, such as pigs, cattle, sheep, goats, and wild ruminants, are susceptible to the virus [1]. FMDV has a single-stranded positive-sense RNA genome that belongs to the genus *Aphthovirus* of the family *Picornaviridae* and is classified into seven serotypes, namely O, A, Asia1, Southern African Territories (SAT)1, SAT2, SAT3, and C [2]. Cross-protection between different serotypes is not possible, and continuous evolution and mutation of the virus greatly hinder treatment [3]. Susceptible animals are infected with FMDV through various transmission routes, including direct and indirect contact, vehicles, and airborne aerosols [4]. Infection with FMDV is followed by a systematic spread, typically forming vesicles in the mouth, tongue, gums, udder, teats, and claws [5].

Inactivated FMDV (iFMDV) antigens are produced from suspension cell culture supernatants of FMDV-infected cells that are chemically inactivated by treatment with binary ethylenimine and purified via ultrafiltration and polyethylene glycol precipitation [6]. Because the iFMDV antigen is poorly immunogenic, formulation with an effective adjuvant is essential for sufficient vaccine-mediated host protection [7]. Although several adjuvants, such as aluminum hydroxide, mineral salts, emulsions, potassium phosphate, saponins, cytokines, polymers, microbial products, and immune-enhancing molecules, have been extensively investigated, only certain types (oil-based adjuvants, such as oil-in-water (O/W), water-in-oil (W/O), and water-in-oil-in-water (W/O/W); aqueous adjuvants, such as aluminum hydroxide and saponin; or a combination of both) are used for field-level applications [8]. Oil-based adjuvants have several advantages over aqueous adjuvants, such as yielding robust and long-term immune responses and being effective vehicles for antigen transport, but they have side effects, including swelling, necrosis, and hemolysis at the injection site [9]. Aluminum hydroxide, currently the most widely used and licensed “gold standard” vaccine adjuvant, has proven to be efficient and safe but has certain limitations, such as poor induction of cell-mediated immunity, especially T-cell response [10]. Saponins induce balanced cell (T helper cell type 1 (Th1) and Th2)-mediated and antibody (B cell)-mediated immune responses [11] but have side effects including cytotoxicity and hemolysis [12]. To address these limitations, it is necessary to identify novel alternatives that can induce robust, long-lasting, and balanced Th1/Th2 immune responses; are safe to use and easy to source; and can be formulated with various antigens.

The integration of small molecule adjuvants has been explored to develop new adjuvants with the advantages mentioned above. Small molecule drugs have played a pivotal role in the pharmaceutical industry, and virtually all traditional drugs and >90% of currently marketed therapeutics are small molecules [13]. These drugs have various benefits, such as high therapeutic impact at low doses, cost effective production, stability, high-quality maintenance without deterioration, exceptional reproducibility, and consistent efficacy [14]. They can be prescribed in oral form, do not have to be injected, and can penetrate cell membranes and reach intracellular targets owing to their small size [15]. These benefits ease experimental applications in new fields and shorten the international drug approval period. Small molecule drugs are optimal therapeutic tools for treating infections caused by bacteria, fungi, and viruses and are excellent vaccine adjuvant candidates with considerable potential for treating and preventing immune diseases through modulation and enhancement of innate and adaptive immunity [16,17].

We explored the small molecule bestatin as an immune-enhancing adjuvant for a novel iFMDV vaccine. Bestatin is a biological response modifier of aminopeptidases found in the culture medium of *Streptomyces olivoreticuli* [18], a natural product and small molecule dipeptide, and it is mainly used as an aminopeptidase (AP)-N inhibitor, immunomodulator, and antitumor drug [19,20]. Bestatin binds to the cell surface and inhibits AP-Ns, such as AP-B and leucine AP [21], and has been investigated for the treatment of several cancers and inflammatory diseases. Bestatin mediates the activation of immune cells through AP inhibition; however, the downstream signaling pathways that bestatin induces through AP inhibition have not been fully elucidated [18,22].

Previous studies on changes in cytokine secretion related to bestatin have shown that bestatin has stimulatory effects on the induction of cytokines, including interleukin (IL)-1 and IL-2, in unstimulated mouse peritoneal macrophages (MΦ) [23], interferon (IFN)γ in mouse splenocytes [24], and IL-6 in unstimulated human peripheral blood mononuclear cells (PBMCs) [25]. However, the precise mechanism by which bestatin modulates cytokine production and secretion remains unclear. In addition, bestatin has been reported to promote cytotoxic T lymphocyte activation [26], antibody production [27], and humoral immune responses [28]. Considering the biological activities and immunomodulatory properties of bestatin, it may be a suitable immunologic adjuvant for targeting immune cell-mediated response; furthermore, owing to its antiviral effects [23] and immune restoration and hematopoietic stimulation properties [29], it is a promising adjuvant candidate for the development of vaccines.

Bestatin is known to exhibit direct and indirect immunomodulatory functions on lymphocytes and monocytes [30]. Additionally, bestatin is an inhibitor of leukotriene A4 hydrolase (LTA4H) and has anticancer effects in colorectal cancer [31]. It also has anticancer effects in gastric cancer by activating the cluster of differentiation 13 (CD13)/NAB1/MAPK pathway [32]. Based on the immunomodulatory function and the anticancer effect of bestatin, we hypothesized that adding bestatin as an adjuvant (immunomodulator) to the FMD vaccine would induce a strong and long-lasting immune response. However, data regarding the efficacy of bestatin as a vaccine adjuvant and its vaccine-mediated immune response are limited.

To address the limitations of current commercial FMD vaccines and design new vaccine candidates against FMDV, the present study evaluated the effectiveness of bestatin as a vaccine adjuvant to enhance early, intermediate-, and long-term immunity by regulating the host’s immune system in mouse (experimental animal) and pig (target animal) models. Additionally, changes in the gene expression of immunoregulatory molecules were observed to identify the mechanism underlying bestatin-mediated innate and adaptive immunity in pigs.

## 2. Materials and Methods

### 2.1. Animals and Ethics Statement

The mouse (experimental animals) and pig (target animals) management method was performed according to methods previously reported [5,33,34]. Mice (C57BL/6, females; 6–7 weeks-old) and pigs (Landrace, 8–9 weeks-old) seronegative for FMDV antibodies were used. This study was conducted with approval from our institutional ethics committee (APQA; IACUC-2022-658) [3,4,34].

### 2.2. Antigen Purification

Purified antigens were prepared from BHK-21 (C-13) (baby hamster kidney cell line; American Type Culture Collection (ATCC), Manassas, VA, USA) cells infected with FMDV type O (O/PAK/44 (O PA2), GenBank Accession No. AY593829.1) and A (A/SKR/YC/2017 (A YC), GenBank Accession No. KY766148.1), according to a modified method as previously described [5,33,34]. Briefly, cytopathic effects (CPEs) were identified 16 h after FMDV infection; the virus was inactivated with 0.003 N binary ethylenimine (BEI; Sigma-Aldrich, St. Louis, MO, USA) twice in 24 h. After confirming that the virus was inactivated via an inactivation confirmation test, the virus was concentrated with polyethylene glycol 6000 (Sigma-Aldrich) [5,35]. Antigen (146S) was purified by sucrose density gradient ultracentrifugation. Then, fractions were collected by puncturing the bottom of the ultracentrifuge tube. To confirm viral inactivation, the BEI-treated supernatant was passed via goat ZZ-R 127 (fetal goat tongue epithelial cell line; ATCC) and BHK-21 cells at least in triplicate [3,4,5].

### 2.3. Murine Peritoneal Exudate Cell (PEC) and Porcine PBMC Isolation

PECs were purified from C57BL/6 mice as previously reported [4,5,34]. Mice were euthanized with CO_2_. The peritoneal cavity was flushed with 5 mL Hanks’ Balanced Salt Solution (HBSS; Hyclone™, Logan, UT, USA). The pelleted PECs-derived peritoneal lavage fluids were resuspended with RPMI 1640 medium (Gibco, Waltham, MA, USA).

PBMCs were purified from pigs as previously described [5,33]. Blood was individually collected, and PBMCs were isolated using Histopaque (Sigma-Aldrich). PBMCs were suspended in HBSS (Gibco). The purified PBMCs were resuspended with RPMI 1640 medium (Gibco) [3,4,34].

### 2.4. Cell Viability Assay

BHK-21, LF-BK (fetal porcine kidney cell line; Plum Island Animal Disease Center, Orient, NY, USA), and ZZ-R 127 cells (2 × 10^4^ cells/well) were treated and cultured for 48 h. Purified PECs and PBMCs (1 × 10^5^ cells/well) were treated and stabilized for 1 h. Then, bestatin (0.625, 1.25, 2.5, or 5 μg/mL; Sigma-Aldrich) was added and allowed to stand for 4 h. Cell viability was measured using an MTS assay (Abcam, Cambridge, MA, USA) following the manufacturer’s recommendations.

### 2.5. Enzyme-Linked Immune Absorbent Spot (ELISpot) Assay

Bestatin (Sigma-Aldrich), with or without antigen-induced IFNγ-secreting cells, was assessed using the ELISpot Plus Kit (Mabtech, Nacka Strand, Sweden) following the manufacturer’s recommendations. PECs or PBMCs (5 × 10^5^ cells/well) were stimulated with 2 μg/mL antigen (O PA2 or A YC) and/or 0.625, 1.25, 2.5, and 5 μg/mL of bestatin sequentially. Antigen and PBS were treated as the positive control (PC) and negative control (NC), respectively. Data were quantified with the ELISpot reader (AID iSpot Reader System; AID Autoimmun Diagnostika GmbH, Straßberg, Germany) [3,4,34].

### 2.6. Early Host Defense in Mice Administered with Bestatin Alone

We determined whether bestatin alone elicited host protection against FMDV infection in mice. The experimental (Exp) group of mice received 100 μg bestatin/100 μL dose. The NC group was administered PBS. Mice (*n* = 5/group) were administered via intramuscular (I.M.) injection (0 dpi (days post-injection (dpi)) and infected with FMDV (100 lethal dose, 50% (LD_50_) of O/VET/2013 or 100 LD_50_ A/Malay/97) by intraperitoneal (I.P.) injection at 3 dpi or 7 dpi. Animals were monitored for up to 7 days post-challenge (dpc).

### 2.7. FMDV Challenge after Immunization with the FMD Vaccine Containing Bestatin in Mice

We performed animal experiments to evaluate the adjuvanticity of bestatin and validated the early protective effects mediated by the FMD vaccine containing bestatin. The PC group vaccine (1 dose/100 μL) formula used in this study was as follows: antigens (O PA2 + A YC, 0.375 + 0.375 μg/dose), 10% Al(OH)_3_, Montanide ISA 206 VG (SEPPIC, Paris, France; 50% *w*/*w*), and 15 μg Quil-A (InvivoGen, San Diego, CA, USA). The Exp group was administered the PC group of vaccines, with the addition of 100 μg bestatin as an adjuvant. Mice were immunized via I.M. injection (0 days post-vaccination (dpv)) and infected with FMDV type O or type A as mentioned in Section 2.6 via I.P. injection at 7 dpv. The NC group was administered PBS [3,4,34]. Animals were monitored until 7 dpc.

### 2.8. Early, Intermediate-, and Long-Term Immune Responses in Mice after Immunization with the FMD Vaccine Containing Bestatin

We evaluated early, intermediate-, and long-term immune responses in mice to determine the adjuvanticity of bestatin. The formula of the vaccine was the same as that mentioned in the previous section. Mice (*n* = 5/group) were immunized with the vaccine via I.M. injection, and blood was collected at 0 and 7 (early term); 28 (intermediate-term); and 56 and 84 dpv (long-term) for structural protein (SP) O and SP A enzyme-linked immunosorbent assay (ELISA) and virus neutralization (VN) tests [3,4,34].

### 2.9. Early, Intermediate-, and Long-Term Immune Responses in Pigs after Immunization with the FMD Vaccine Containing Bestatin

To assess the adjuvanticity and efficacy of bestatin in inducing early, intermediate-, and long-term immune responses in pigs, experiments were conducted as previously described [5,33]. The PC group vaccine (1 dose/1 mL) formula used in this study was as follows: antigens (O PA2 + A YC, 15 + 15 μg/dose), 10% Al(OH)_3_, Montanide ISA 206 VG (SEPPIC; 50% *w*/*w*), and 150 μg Quil-A (InvivoGen). The Exp group was administered the PC group of vaccines, with the addition of 1 mg bestatin as an adjuvant. The NC group was administered PBS. Blood was collected at 0, 7, and 14 (early term); 28 and 42 (intermediate-term); and 56 and 84 dpv (long-term) for serological analysis (ELISA and VN test) and real-time quantitative PCR (RT-qPCR) [3,4,34].

### 2.10. SP ELISA

To measure SP antibodies in serum samples, the FMDV type O- (VDPro^®^ kit; Median Diagnostics, Chuncheon, Republic of Korea) and A-specific ELISA (PrioCheck^TM^; Prionics AG, Schlieren, Switzerland) kits were used following the manufacturer’s recommendations. Absorbance was detected using a spectrophotometer (Hidex, Turku, Finland) at 450 nm [3,4,34]. Data were converted to the percent inhibition (PI) value. The animals were considered antibody-seropositive if the PI value was ≥40% for the VDPro^®^ FMDV kit or ≥50% for the PrioCheck^TM^ FMDV kit. 

### 2.11. VN Test

A VN test was performed following the WOAH manual [5]. The sera were inactivated and prepared as two-fold serial dilutions. VN titers in the sera were analyzed using a VN test for O PA2 and A YC in LF-BK cells [3,4,34,36].

### 2.12. ELISA for the Detection of Isotype-Specific Antibodies (IgG, IgA, and IgM)

Porcine IgG, IgA, and IgM (Bethyl Laboratories/Fortis Life Sciences, Montgomery, TX, USA) were detected on the sera via isotype ELISA following the manufacturer’s recommendations. Data were obtained using a spectrophotometer (Hidex) at 450 nm [3,4,34].

### 2.13. RNA Extraction, Complementary DNA (cDNA) Synthesis, and RT-qPCR

To elucidate the mechanism of immune response elicited by vaccines with bestatin, an experiment was conducted as previously described [5,33,34]. Total RNA was extracted using QIAzol and the RNeasy Mini Kit (QIAGEN, Valencia, CA, USA) following the manufacturer’s recommendations. cDNA was synthesized using the GoScript Reverse Transcription System (Promega, Madison, WI, USA) following the manufacturer’s recommendations. The synthesized cDNAs were amplified by RT-qPCR using iQ SYBR Green Supermix (Bio-Rad, Hercules, CA, USA). Data were normalized to *HPRT* (reference gene) levels and expressed as relative ratios [3,4,34]. The list of primers was presented in Table S1.

### 2.14. Statistical Analysis

Data were presented as the mean ± standard error. Between-group differences were compared by using one-way or two-way analysis of variance with Tukey’s or Dunnett’s post–hoc tests. The survival rate was evaluated using Kaplan–Meier survival curves. GraphPad Prism software version 10.0.2 (San Diego, CA, USA) was used.

## 3. Results

### 3.1. Bestatin Induces Robust IFNγ Secretion in Murine PECs and Porcine PBMCs In Vitro

**Figure 1:**
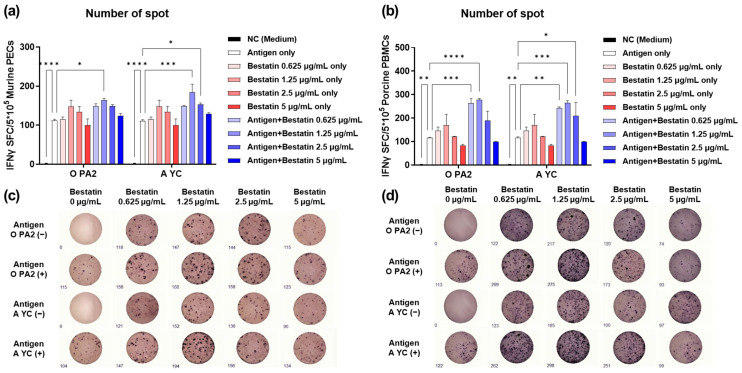
Foot-and-mouth virus (FMDV) antigen (O PA2 or A YC) was treated with bestatin to induce interferon (IFN)γ secretion in murine peritoneal exudate cells (PECs) and porcine peripheral blood mononuclear cells (PBMCs). IFNγ secretion was assayed to evaluate the innate immune responses induced by bestatin with or without inactivated FMDV (iFMDV) antigens using the enzyme-linked immune absorbent spot (ELISpot) assay. (**a**–**d**) Mean number of IFNγ spot-forming cells (SFCs) stimulated under different conditions on PECs (**a**); mean number of IFNγ SFCs stimulated under different conditions on PBMCs (**b**); ELISpot representative images of IFNγ activated with negative control (NC), antigen (O PA2 or A YC) only, bestatin only, and antigen with bestatin for each concentration on PECs (**c**); ELISpot representative images of IFNγ activated with NC, antigen (O PA2 or A YC) only, bestatin only, and antigen with bestatin for each concentration on PBMCs (**d**). Data are presented as mean ± standard error of the mean (SEM) of SFCs from triplicate measurements (*n* = 3/group). * *p* < 0.05, ** *p* < 0.01, *** *p* < 0.001, and **** *p* < 0.0001 (one-way analysis of variance (ANOVA) followed by Tukey’s test).

Prior to measuring bestatin-induced IFNγ secretion, cytotoxicity by bestatin treatment was confirmed using isolated murine PECs and porcine PBMCs as well as BHK-21, LF-BK, and ZZ-R susceptible to FMDV. Bestatin at a concentration of 0–5 μg/mL did not show cytotoxicity in these cells (Figure S1a–e). To estimate bestatin with or without an antigen-induced cellular immune response, we confirmed that bestatin, iFMDV (type O and A) antigen, and bestatin with iFMDV (type O and A) antigen mediated IFNγ secretion via an in vitro ELISpot assay using PECs isolated from naive mice and PBMCs isolated from porcine whole blood. As shown in Figure 1a,c (murine PECs) and Figure 1b,d (porcine PBMCs), although no significant differences were observed between the two groups, a concentration of 1.25 μg/mL of bestatin was optimal for inducing IFNγ secretion in both the bestatin-only and bestatin-plus-antigen groups. In murine PECs, the average number of IFNγ-secreting cell spots in the group treated with O PA2 or A YC antigens plus 1.25 μg/mL bestatin was higher than that in the group treated with antigen only (*p* < 0.05 and *p* < 0.001, respectively) (Figure 1a,c). In porcine PBMCs, the average number of spots of IFNγ-secreting cells in the group treated with O PA2 or A YC antigen plus 1.25 μg/mL of the bestatin was higher than that in the group treated with antigen only (*p* < 0.0001 and *p* < 0.001, respectively) (Figure 1b,d). These results show that bestatin could induce an innate cell-mediated and Th1-type immune response by enhancing IFNγ secretion. In addition, when iFMDV antigen (type O and A) and bestatin were co-administered, IFNγ secretion increased, thus indicating the potential of bestatin as an adjuvant (immunostimulant).

### 3.2. Inactivated FMD Vaccine Containing Bestatin Elicits Potent Protective Effects in the Early Stages of FMDV Infection in Mice

Before evaluating the host defense by the vaccine containing bestatin, we confirmed whether the host was protected against viral infection by administration with bestatin alone. In the group that was administered bestatin alone, all mice infected with FMDV type O and A at 3 dpi or 7 dpi, respectively, died; we also confirmed that bestatin alone did not elicit the host defense (Figure S2a–i).

**Figure 2:**
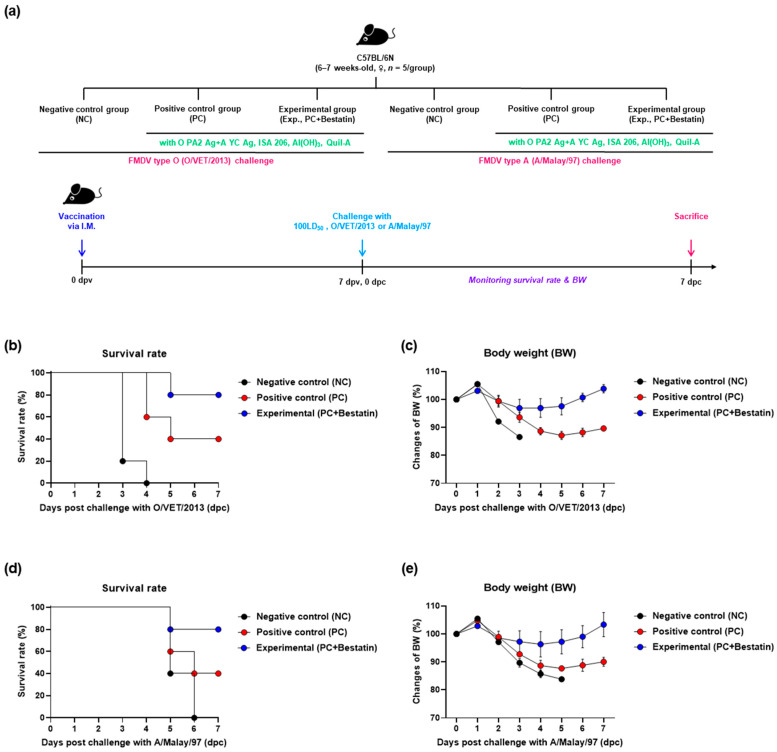
FMDV vaccine containing bestatin showed favorable vaccine efficacy and conferred protective effects on mice. C57BL/6 mice (6–7 weeks-old, *n* = 5/group) were observed for mortality for 7 days, and protection of each group was represented by Kaplan–Meier survival curves. (**a**–**e**) Experimental workflow (**a**); survival rates post-challenge with O/VET/2013 (**b**) and A/Malay/97 (**d**), respectively; changes in body weight post-challenge with O/VET/2013 (**c**) and A/Malay/97 (**e**), respectively. Data are presented as mean ± SEM of triplicate measurements (*n*  =  5/group). Abbreviations: dpv, days post-vaccination; I.M., intramuscular injection; LD_50_, lethal dose, 50%.

To determine the protective effect of the test vaccine containing bestatin against FMDV infection in mice, experiments were performed according to the strategy described in Figure 2a. Mice in the Exp group vaccinated with the test vaccine plus bestatin showed an 80% survival rate against O/VET/2013 and A/Malay/97 (Figure 2b,d). There was no significant change in the body weight in the Exp group (Figure 2c,e). In the PC group that received the test vaccine without bestatin, the survival rate against both FMDV types O and A was 40%, and weight loss increased by more than 10% at 5 dpc for both virus types. In the NC group, the survival rate was 0% at 4 and 6 dpc in FMDV type O- and A-infected groups, respectively.

### 3.3. Bestatin Induces Early, Intermediate-, and Long-Term Immunity in Mice and Pigs

**Figure 3:**
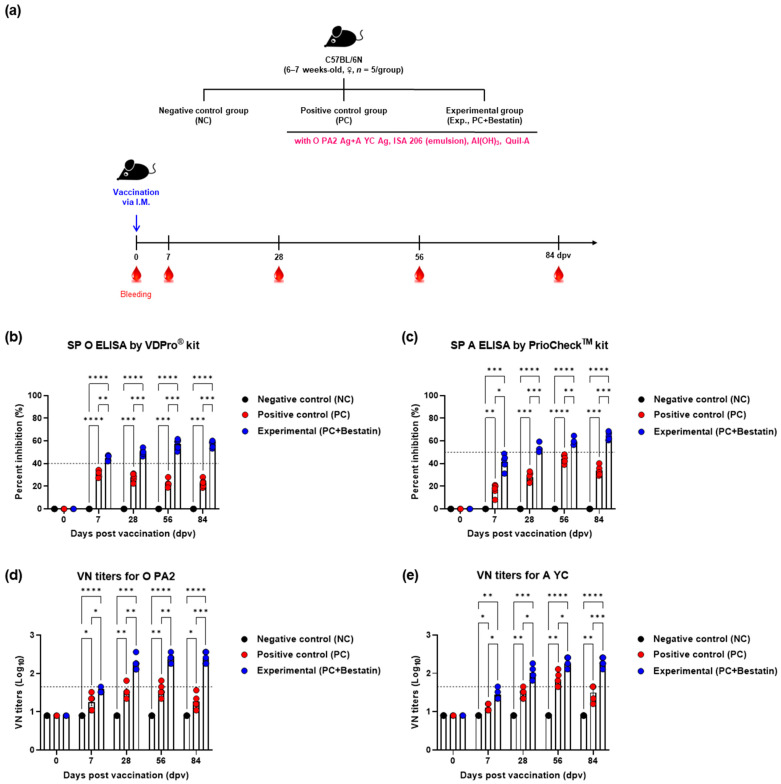
FMDV vaccine containing bestatin upregulated early, intermediate-, and long-term immune responses in mice. C57BL/6 mice (6–7 weeks-old, *n* = 5/group) were vaccinated via I.M. injection. (**a**–**e**) Experimental workflow (**a**); antibody titers using SP ELISA type O (**b**) or type A (**c**); VN titers for O PA2 (**d**) or A YC (**e**) using the VN test. Data are presented as mean ± SEM of triplicate measurements (*n* = 5/group). * *p* < 0.05, ** *p* < 0.01, *** *p* < 0.001, and **** *p* < 0.0001 (two-way ANOVA with Tukey’s test).

To determine the effect of the test vaccines containing bestatin on adaptive immune response, we assessed early, intermediate-, and long-term immunity and memory response to the test vaccines with and without bestatin in mice (Figure 3a). We assessed antibody titers (using VDPro^®^ and PrioCheck^TM^ kits, respectively) and VN titers via VN tests and compared the results yielded by groups that received vaccines with or without bestatin (Figure 3b–e).

Antibody titers in mice were higher in the Exp group than those in the PC and NC groups at all time points from 7 to 84 dpv (Figure 3b,c). The VN titers for O/PKA/44/2008 (O PA2) and A/SKR/YC/2017 (A YC) homologous viruses for the O PA2 and A YC antigens were higher in the Exp group than those in the other groups (PC and NC) from 7 to 84 dpv (Figure 3d,e).

To evaluate the induction of humoral immunity by the bivalent iFMDV vaccine with bestatin in pigs, we monitored the induction of early, intermediate-, and long-term immunity in FMD-seronegative pigs.

**Figure 4:**
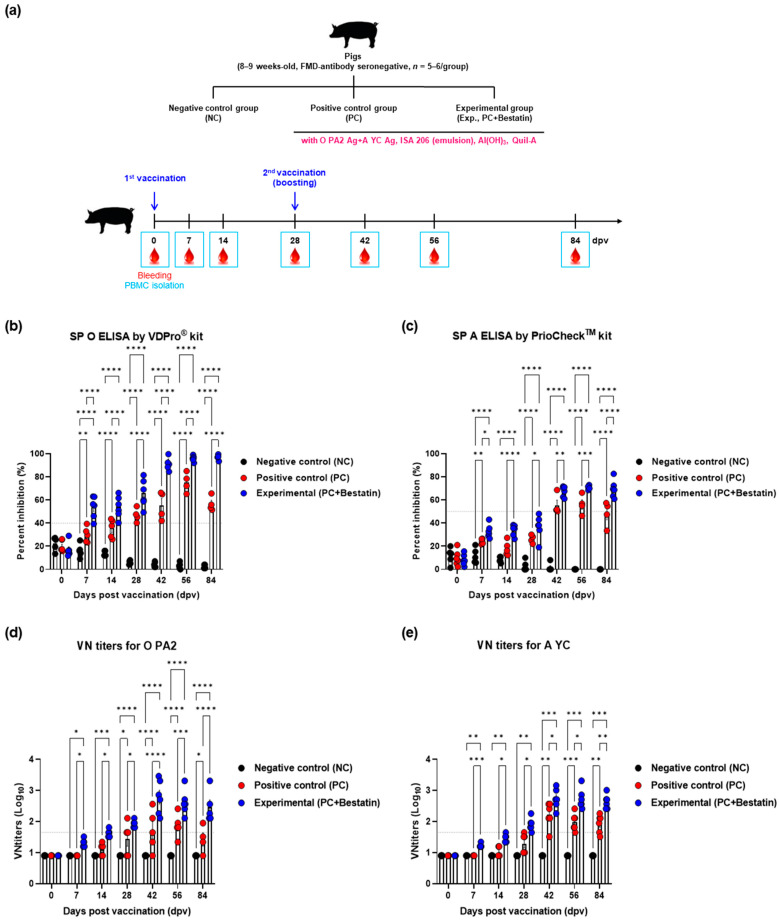
FMDV vaccine containing bestatin upregulated the early, intermediate-, and long-term immune response in pigs. For the pig experiments, FMDV (O and A) antibody-seronegative animals (8–9 weeks-old, *n* = 5–6/group) were used. (**a**–**e**) Experimental workflow (**a**); antibody titers using SP ELISA type O (**b**) or type A (**c**); VN titers for O PA2 (**d**) or A YC (**e**) using the VN test. Data are presented as mean ± SEM of triplicate measurements (*n* = 5–6/group). * *p* < 0.05, ** *p* < 0.01, *** *p* < 0.001, and **** *p* < 0.0001 (two-way ANOVA with Tukey’s test).

FMD-seronegative pigs were vaccinated and after 28 dpv, a booster vaccine was administered. Blood collection and serological tests were performed according to Figure 4a. When the bivalent vaccine containing bestatin was administered to pigs, the antibody titer (assessed using SP O ELISA) increased compared with that in the PC group during the first vaccination period (*p* < 0.0001, 7 dpv) and was maintained until 14 and 28 dpv (*p* < 0.0001, Figure 4b). After the second vaccination at 28 dpv, the Exp group showed significantly higher antibody titers at 42, 56, and 84 dpv than those in the PC group (*p* < 0.0001, Figure 4b). The rate of increase of the antibody titer assessed using SP A ELISA was lower than that assessed using SP O ELISA. Nevertheless, at 7 dpv, the antibody levels in the Exp group were higher than those in the PC group, and seropositivity was observed until 84 dpv (*p* < 0.0001, *p* < 0.001, *p* < 0.01, and *p* < 0.05; Figure 4c). In the PC group, antibody titers reached seropositivity after 28 dpv, decreased at 56 dpv, and eventually changed to seronegativity at 84 dpv (Figure 4c).

VN titers showed similar results to SP ELISA. As a result, VN titers were higher in the Exp group than those in PC group from 7 to 84 dpv (*p* < 0.0001, *p* < 0.001, *p* < 0.01, and *p* < 0.05; Figure 4d,e).

**Figure 5:**
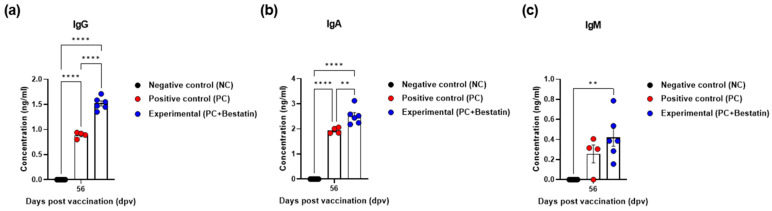
Immune responses mediated by the FMDV vaccine with bestatin measured using the levels of immunoglobulin subtypes IgG, IgA, and IgM in pigs. For the pig experiments, FMDV type O and A antibody-seronegative animals (8–9 weeks-old, *n* = 5–6/group) were used. Single-dose vaccination was administered I.M. twice at 28-day intervals. (**a**–**c**) IgG (**a**); IgA (**b**); and IgM (**c**) concentrations. Data are presented as mean ± SEM of triplicate measurements (*n* = 5–6/group). ** *p* < 0.01, and **** *p* < 0.0001 (two-way ANOVA with Tukey’s test).

To evaluate the effect of the bivalent iFMDV vaccine with bestatin on the production of porcine total immunoglobulin subtypes (IgG, IgA, and IgM), we performed an immunoassay using serum from pigs immunized with the FMD vaccine containing bestatin with the strategy described in Figure 4a. As a result, at 56 dpv, the concentrations of IgG and IgA were higher in the Exp group than those in the PC group (*p* < 0.0001 and *p* < 0.01, respectively). No difference was observed between the IgM concentrations in Exp and PC groups (Figure 5a–c).

### 3.4. Bestatin Induces Robust and Long-Lasting Immune Responses by Enhancing the Expression of Immunomodulatory Molecules

**Figure 6:**
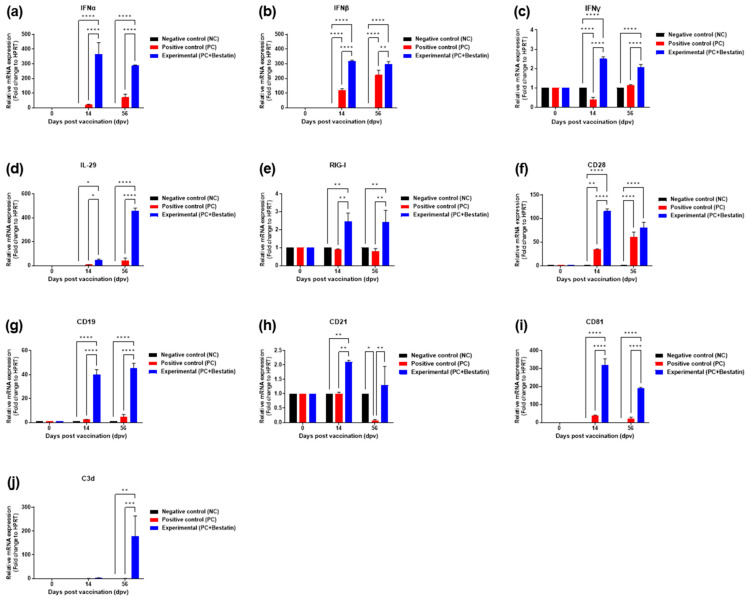
FMDV vaccine with bestatin induced the immunomodulatory gene expression in porcine PBMCs. In Figure 4a, PBMCs isolated from the whole blood of vaccinated pigs (*n*  =  5–6/group) were used for real-time quantitative PCR. Gene expression levels were normalized to *HPRT* levels and are presented as relative ratios compared with control levels. (**a**–**j**) Gene expression of IFNα (**a**); IFNβ (**b**); IFNγ (**c**); IL-29 (**d**); RIG-I (**e**); CD28 (**f**); CD19 (**g**); CD21 (**h**); CD81 (**i**); and C3d (**j**). Data are presented as mean ± SEM of triplicate measurements (*n* = 5–6/group). * *p* < 0.05, ** *p* < 0.01, *** *p* < 0.001, and **** *p* < 0.0001 (one-way ANOVA followed by Tukey’s test).

To elucidate the molecular mechanisms of and robust long-lasting immune response to the iFMDV vaccine containing bestatin, real-time qPCR was performed using porcine PBMCs at the 14 and 56 dpv sampling points (Figure 4a). The results showed changes in the immunomodulatory gene expression of cytokines IFNα, IFNβ, IFNγ, and IL-29; RIG-I; co-stimulatory molecules (CD28, CD19, CD21, and CD81); and complement component 3d (C3d), which are related to the induction of cellular and humoral immune responses. The expression levels of IFNα and IFNβ, which are type I IFNs, were higher in the Exp group than those in the PC group at 14 dpv (*p* < 0.0001 for both groups) and 56 dpv (*p* < 0.0001 and *p* < 0.01, respectively) (Figure 6a,b). The expression levels of IFNγ and type II IFNs were higher in the Exp group than those in the PC group at 14 and 56 dpv (*p* < 0.0001) (Figure 6c). The expression levels of IL-29 in the Exp group were higher than those in the PC group at 14 dpv (*p* < 0.05) and 56 dpv (*p* < 0.0001) (Figure 6d). The expression levels of RIG-I, a cytosolic pattern recognition receptor (PRR) responsible for type I IFNs, were higher in the Exp group than those in the PC group at 14 and 56 dpv (*p* < 0.01 for both groups) (Figure 6e). The expression levels of co-stimulatory molecules were significantly higher in the Exp group than those in the PC group. At 14 dpv, the expression levels of CD28 (Figure 6f), CD19 (Figure 6g), CD21 (Figure 6h), and CD81 (Figure 6i) differed significantly between the Exp and PC groups (*p* < 0.0001, *p* < 0.0001, *p* < 0.01, and *p* < 0.0001, respectively). At 56 dpv, significant differences were observed between the expression levels of CD19, CD21, and CD81 for the Exp and PC groups (*p* < 0.0001, *p* < 0.01, and *p* < 0.0001, respectively). Notably, at 56 dpv, C3d expression was significantly higher in pigs vaccinated with bestatin than that in the PC group (*p* < 0.001) (Figure 6j).

## 4. Discussion

The wide species tropism, epidemiological complexity at the interface between domestic and wild animals, human migration, various modes of transmission, wide genetic diversity, rapid replication rates, and high infection rates of FMDV complicate its control [37]. Therefore, the infection of susceptible animals can result in significant economic and financial losses associated with infection control and elimination. The control of this disease necessitates the establishment of several measures, including strict sanitary measures, migration control, restrictive policies, and biosecurity measures. Vaccination is the most effective prevention strategy for FMDV. An ideal FMD vaccine is safe and cost-effective, can be mass-produced, induces a sufficient protective immune response upon administration of a single dose, produces rapid and long-lasting multivalent immunity, and protects against FMDV serotypes and sub-serotypes (topotypes) [37].

Adjuvants have been developed to combat several infectious diseases by enhancing the immune response induced by vaccines [38]. For inactivated vaccines, adjuvants are required to enhance the delivery of vaccine antigens and stimulate PRRs that are difficult to induce with antigens alone [39]. Therefore, research aimed at identifying and developing novel and powerful adjuvants such as cytokines [40], co-stimulator molecules [41], and other immunostimulators such as PRR ligands (agonists) [42] that enhance the efficacy of FMD vaccines is ongoing.

As vaccine adjuvants, small molecule drugs have many advantages. Most small molecule drugs are composed of simple chemical structures, resulting in low molecular weight and high stability. These properties allow small molecule drugs to easily cross cell membranes and stimulate intracellular proteins that other adjuvants cannot [14]. There are numerous candidate groups and data on small molecule drugs that can modulate immunity, which are applicable to FMD vaccines and other infectious diseases.

Bestatin, a small molecule dipeptide that can be used orally, is a potent biological response modifier and inhibitor of APs with immunomodulatory and antitumor functions [20]. In addition to the antiviral and antitumor effects exerted by bestatin as an AP inhibitor, the aforementioned studies have reported improvement in immunocompetent cell functions, changes in cellular activity and cytokine production, antibody production, and stimulation of the humoral immune response, indicating that bestatin is a suitable candidate for a vaccine adjuvant. Sasaki et al. [23] proposed bestatin as an effective adjuvant when designing an HIV-1 DNA vaccine. Despite its various immunomodulatory functions, few studies have used bestatin as a vaccine adjuvant. Therefore, in this study, bestatin was selected as a vaccine adjuvant candidate and formulated into the iFMDV vaccine to facilitate the evaluation of its immunomodulatory effects in mice and pigs. In this study, bestatin-mediated cytotoxicity was confirmed prior to in vitro studies, but no cytotoxicity was observed at concentrations of 0–5 μg/mL (Figure S1); hence, the following experiment was performed.

First, we evaluated the potential of bestatin alone and in combination with iFMDV (types O and A) antigens on IFNγ induction via an in vitro ELISpot assay using mouse PECs and porcine PBMCs. The addition of bestatin increased IFNγ secretion in each Exp group. IFNγ is a type II IFN and plays a pivotal role in T-cell-mediated cellular immune responses [43,44]. IFNγ is also secreted by natural killer (NK), CD4^+^ T, and CD8^+^ T cells. IFNγ stimulates type I IFN production and orchestrates the immune response to viral infection [45], promotes antiviral activity via NK cells, and enhances antigen presentation and phagocytosis of MΦ [46]. PECs are suitable to study cellular immune responses in antigen-presenting cells, including dendritic cells (DCs), MΦ, monocytes, and unconventional T cells, including γδ T cells, invariant NK T cells, and mucosal-associated invariant T cells [3]. PBMCs consist of lymphocytes (NK, T, and B cells) and monocytes and can be used to assess cell-mediated immunity generally or via antigen-specific stimulation [47]. Our data show that bestatin induces a cellular immune response in murine PECs and porcine PBMCs through IFNγ secretion, confirming that bestatin effectively induces Th1-cell-mediated cellular immune responses as well as NK-cell-mediated innate immune responses. In addition, the highest efficacy of bestatin observed at a concentration of 1.25 μg/mL indicates the importance of additive concentrations and offers indirect evidence of the immunomodulatory functions of bestatin (Figure 1). Given the significant levels of IFNγ induced by bestatin in the ELISpot assay, we speculated that the rapid and robust cellular immune response induced by bestatin would protect the host against FMDV by clearing the virus early. Before performing experiments on the target animals (pigs), we investigated the protective effects of iFMDV vaccines with or without bestatin against the infection of FMDV types O and A in mice. The early stage of vaccination effectively protected the mice against FMDV infection. A comparison between the survival rates and changes in the body weights of mice vaccinated and exposed to the FMDV showed that the vaccine adjuvanted with bestatin was the most effective for protection against FMDV infection (Figure 2). This may be attributed to an increased cellular immune response resulting from IFNγ secretion and related downstream signals. Moreover, host defense with administration with bestatin alone was not observed, demonstrating that the addition of bestatin to a vaccine as an adjuvant can elicit an immune-enhancing effect (Figure S2).

We then validated the early, intermediate-, and long-term immunity elicited by the iFMDV vaccine containing bestatin in mice and pigs. To determine the efficacy of bestatin, a bivalent iFMDV (containing O PA2 + A YC antigen) vaccine with or without bestatin was administered to mice and pigs. SP ELISA tests (type O and A) revealed that the antibody titers were higher in the group that received the vaccine with bestatin than those in the group that received the vaccine without bestatin in the experimental (mice) and target animals (pigs) (Figure 3 and Figure 4). Notably, the antibody titers in the PC group decreased after 56 dpv, whereas those in the vaccine-plus-bestatin group remained high until 84 dpv, indicating the long-lasting effect of bestatin.

To identify neutralizing antibodies against viral infection, VN titers were measured for each Exp group. Vaccination-induced VN titer levels of ≥1.74 (Log_10_) have been reported to protect against FMDV infection [48]. According to this study, an FMD vaccine containing bestatin will protect the host from FMDV infection from 28 dpv onwards until at least 84 dpv. The Korean FMD Vaccine Evaluation Criteria stipulate that the host can be protected against FMD infection only when the VN antibody titers by vaccination exceed 1.65 (Log_10_) [4]. Thus, according to Korean FMD Vaccine Evaluation Criteria, it can be assumed that the FMD vaccine containing bestatin will protect the host after 14 dpv. Taken together, FMD vaccines containing bestatin achieve short-term and long-term maintenance of VN antibody titers at levels that induce host defenses (Figure 3 and Figure 4). We previously reported a strong relationship between VN titers and protection against FMDV in pigs [49]; therefore, the iFMDV vaccine containing bestatin is expected to provide sufficient host protection for a relatively long duration.

Additionally, the measurement of antigen-specific (SP-nonspecific) antibody titers, including IgG, IgA, and IgM in serum at 56 dpv, showed that the iFMDV vaccine containing bestatin effectively induced IgG and IgA levels (Figure 5). This provides conclusive evidence that the vaccines containing bestatin could increase the antibody titers, VN titers, and total IgG and IgA titers and maintain them for a long time, demonstrating that test vaccines containing bestatin elicit long-lasting immune responses.

To define the mechanism underlying the robust and long-lasting immune response induced by vaccine supplementation with bestatin, gene expression levels of IFNα, IFNβ, IFNγ, IL-29, RIG-I, CD28, CD19, CD21, CD81, and C3d were quantified using samples collected at 14 and 56 dpv. As a result, significantly higher gene expression levels were observed in the Exp group than those in the control group for each gene, which suggests that these genes are involved in the immunological mechanism responsible for the strong immune response triggered by bestatin (Figure 6).

IFNs belong to a family of cytokines that stimulate antiviral activity in the host and are divided into three groups, namely types I, II, and III. Type I IFNs are secreted during viral infection and exhibit antiviral activity via interaction with IFNα/β receptors expressed on most immune cells [50]. IFNα and IFNβ are secreted by numerous cells, including lymphocytes, MΦ, fibroblasts, endothelial cells, and osteoblasts. Type I IFNs inhibit viral spread to surrounding cells and promote the formation of an immunological memory by promoting innate and adaptive immune responses [51].

PRRs activate innate immunity by recognizing invaders [52]. RIG-I is a PRR involved in the IFN I and IFN III responses and is present in most cells of the immune system [53]. IL-29 is an antiviral cytokine belonging to the type III IFN family and is also termed IFNλ [54]. IFNλ is secreted by MΦ, DCs, or mastocytes and plays a crucial role in the immune response to viral infection [55]. IFNλ primarily inhibits viral infection in the primary cells of epithelial origin and hepatocytes [56]. Type I IFNs promote antiviral immunity and link innate and adaptive immune responses [57]. IFNλ signaling differs in many respects from IFNα and IFNβ signaling, especially in neutrophils [58]. However, numerous recent studies have reported a functional link between IL-29 levels and adaptive immunity. IL-29 receptor transcripts have been detected in human naive, memory B, and plasmacytoid DCs; IL-29 treatment elicited signal transducer and activator of transcription 1 (STAT1) phosphorylation and IFN-stimulated gene expression in human B cell and plasmacytoid DCs [59] and promoted the expression of co-stimulatory molecules and chemokine synthesis [60]. In addition, IL-29 modulates cytokine production in other cells such as monocytes and MΦ and enhances the responsiveness of MΦ to IFNγ by increasing the expression of IFNγ receptor 1. Through stimulation of MΦ, IL-29 can also indirectly affect NK cells, stimulate T-cell polarization towards the Th1 phenotype, and mediate the B-cell response to IL-29 [61]. Collectively, these findings suggest that IL-29 affects the release of inflammatory cytokines from T cells and regulates T-cell differentiation and B-cell function.

Moreover, Ye et al. [58] reported that IL-29 is a potent mediator of the adaptive immune response that is selectively activated in the respiratory mucosa. IL-29 elicits potent antiviral effects similar to type I IFNs. The main difference between IL-29 and type I IFNs is that type I IFNs have a short-term effect, whereas IL-29 has a long-lasting effect. These reports indicate that the significant increase in IL-29 following treatment with bestatin is one of the major causal factors of robust and long-term immunity. IL-29 activates DCs via the synthesis of thymic stromal lymphopoietin, thereby promoting the maturation of CD8^+^ T cells and T follicular helper cells, which results in enhanced secretion of virus-specific IgG and IgA. Ye et al. [62] also reported that when infected with influenza virus, IFNλ receptor-deficient mice had significantly attenuated CD8^+^ T-cell activity and lowered virus-specific IgG and IgA levels compared with wild-type mice. These findings are consistent with our results showing increased total IgG and IgA antibody titers and provide evidence for the potent and long-lasting effect of the elevated secretion of bestatin-mediated IL-29.

CD28 is one of the proteins expressed in T cells that provides co-stimulatory factors and is required for T cell activation and survival [63]. T-cell stimulation through CD28 can be a potent signal to produce various ILs. The significant increase in CD28 provides evidence of an enhanced cellular immune response by bestatin. Gauld et al. [64] reported that activation of CD19 induces a humoral immune response by stimulating both antigen-dependent and antigen-independent pathways in B cells. CD21, also known as the C3d receptor and complement receptor type 2 (CR2), is expressed on B-cell surfaces, allowing the complement system to play a pivotal role in B-cell differentiation [65]. CD21 also forms a complex with CD19 and CD81 (target of an antiproliferative antibody, TAPA-1) on mature B cells [66]. During B-cell activation, CD81 orchestrates the localization of CD19, contributing to B-cell-mediated signaling [67,68]. Therefore, the strong and long-term immunity due to the increased expression of CD19, CD21, and CD81 may have been caused by bestatin. As shown in this study, the additive result of the increase in C3d gene expression at 56 dpv indicates another specificity of bestatin.

The complement system, a major component of innate immunity, is known to not only be involved in inflammation but also play a role in strengthening the adaptive immune response [69]. The complement system covalently binds C3d to microbial antigens, which bind to CR2 on B lymphocytes, significantly enhancing the adaptive immune response to those antigens [70]. As a molecular adjuvant, complement C3d has been reported to be a bridge between innate and adaptive immunity [71]. Previous studies have attempted to increase B-cell signaling and induce both cellular and humoral immunity against various antigens using C3d [72,73,74]. C3d binds antigens, enhances the immune response to fused antigens, and contributes to maintaining memory B cells [75].

Overall, the diverse mechanisms through which bestatin functions as an adjuvant are as follows. The increased secretion of type I IFNs (IFNα and IFNβ) and II IFNs (IFNγ) from NK cells and MΦ, among others, following the addition of bestatin induced immunomodulatory effects, including activation of various immune cell types (NK cells, MΦ, CD4^+^ T cells, CD8^+^ T cells, etc.), trafficking, differentiation, and direct intracellular antiviral activities through a positive feedback mechanism. The increased secretion of type III IFNs (IL-29) from MΦ and DCs also induces immunomodulatory effects on various immune cells (MΦ, DCs, CD4^+^ T cells, and CD8^+^ T cells). RIG-I expression is also increased by the addition of bestatin, thereby inducing stronger cellular and humoral immunity. Additionally, there was increased expression of the B-cell co-receptor complex (CD19–CD21–CD81) and C3d-induced B-cell capability-enhancing effects, such as B-cell activation, maturation, increased life span, and maintenance of memory B cells. Increased secretion of IL-29 robustly enhances antigen-specific IgG and IgA production. IL-29 induces DC activation, thereby enhancing CD8^+^ T-cell maturation in the draining lymph nodes and promoting germinal center reactions through effects on T follicular helper cells, resulting in a more potent and long-lasting cellular and humoral immunity.

To date, the application of small molecules for the treatment of viral infections is limited to their antiviral effects. Antiviral drugs generally have a short half-life, which has the disadvantage of short-term effects. In this study, bestatin, which has been reported to have immunomodulatory and therapeutic effects when used as an immunochemotherapy adjuvant after cancer surgery, was used as a vaccine adjuvant to prevent FMD. The FMD vaccine containing bestatin induced a potent and long-lasting immune response by simultaneously inducing cellular and humoral immune responses. Although mechanistic analyses to elucidate the role of bestatin as an adjuvant in the FMD vaccine were limited and performed partially on some genes in this study, further studies are needed to identify the comprehensive mechanisms involved in bestatin-mediated immune responses. This study demonstrates the feasibility of bestatin as an adjuvant for FMD vaccines; thus, our study can be used as a blueprint for rational vaccine design and development strategies to prevent and control other difficult-to-treat animal diseases.

## 5. Conclusions

Bestatin with iFMDV (type O and A) antigens enhanced the cellular immune response by increasing the secretion of IFNα, IFNβ, IFNγ, RIG-I, IL-29, and CD28 and increased the secretion of the B-cell co-receptor complex (CD21–CD19–CD81) and C3d. This in turn enhanced and prolonged the response of B cells to antigens, thereby inducing robust and long-lasting cellular and humoral immune responses. These results suggest that our novel viral vaccine formulation containing bestatin can induce a mixed and balanced Th1/Th2 immune response. Furthermore, the vaccination cycle can be extended by improving short-term immunity, which is a major disadvantage of the iFMDV vaccine currently in use. The results of the enhanced efficacy of the iFMDV vaccines by modulating the host’s innate and adaptive immune responses provide clues to vaccine strategies to prevent not only FMD but also COVID-19 and other dangerous viral diseases.

## Data Availability

All data supporting the findings of this study are available from the corresponding author upon reasonable request.

## Supplementary Materials

The following supporting information can be downloaded at https://www.mdpi.com/article/10.3390/vaccines11111690/s1, Table S1: List of primer sequences for real-time quantitative PCR; Figure S1: Cytotoxicity of bestatin measured in BHK-21, LF-BK, ZZ-R, murine peritoneal exudate cells (PECs), and porcine peripheral blood mononuclear cells (PBMCs) via cell viability assay; Figure S2: Assessment of induction of host defense against foot-and-mouth disease virus (FMDV) infection 3 or 7 days post-infection with bestatin alone in mice.
