# Supplementary material for: Bestatin, A Pluripotent Immunomodulatory Small Molecule, Drives Robust and Long-Lasting Immune Responses as an Adjuvant in Viral Vaccines
Source: Vaccines (Basel). 2023 Nov 4;11(11):1690. doi: 10.3390/vaccines11111690 (PMC10675184; doi:10.3390/vaccines11111690)

Supplementary Table legends

Table S1. List of primer sequences for qRT-PCR.

Supplementary Figure legends

Figure S1. Cytotoxicity of bestatin measured by cell viability assay in BHK-21, LF-BK, ZZ-R, murine PECs and porcine PBMCs.

(**a**–**e**) Cell viability of BHK-21 (**a**); LF-BK (**b**), ZZ-R cells (**c**); murine PECs (**d**); and porcine PBMCs (**e**).

Data have been represented as the mean ± SEM of triplicate measurements (*n* = 3/group). Statistical analyses were performed using one-way ANOVA with Dunnett's *post hoc* test.

Figure S2. Bestatin alone-mediated host defense in early stage of FMDV infection on mice.

C57BL/6 mice (6–7 weeks-old, *n* = 5/group) were administered intramuscularly a bestatin alone. Mice were challenged with FMDV O (100 LD_{50} O/VET/2013) or FMDV A (100 LD_{50} A/Malay/97) at 3 or 7 days post-injection (dpi) using an intraperitoneal injection. Survival rates and body weights were monitored for 7 days post-challenge (dpc) with the respective viruses. (**a**–**i**) experimental workflow (**a**); survival rates in 3 dpi challenged group with O/VET/2013 (**b**) or A/Malay/97 (**c**); changes in body weight 3 dpi challenged group with O/VET/2013 (**d**) or A/Malay/97 (**e**); survival rates in 7 dpi challenged group with O/VET/2013 (**f**) or A/Malay/97 (**g**); and changes in body weight 7 dpi challenged group with O/VET/2013 (**h**) or A/Malay/97 (**i**). Data are presented as mean ± SEM of triplicate measurements (*n* = 5/group).

Table S1

Target	Forward/Reverse	Sequence (5-3)	Length(mer)
IFNα	IFNα F	CATCTGCTCTCTGGGCTGTG	20
	IFNα R	TGAGGGGATCCAAAGTCCCT	20
IFNβ	IFNβ F	TGCAACCACCACAATTCCAGA	21
	IFNβ R	GGTTTCATTCCAGCCAGTGC	20
IFNγ	IFNγ F	GCCATTCAAAGGAGCATGGAT	21
	IFNγ R	CTGATGGCTTTGCGCTGGAT	20
IL-29	IL-29 F	GCTACAGCTTGGATCGTGGT	20
	IL-29 R	TCAGGTCCCTTACGAGGAGG	20
RIG-I	RIG-I F	GCACCTCATACTTACAGCCCA	21
	RIG-I R	CCACAACCAGTAGGAGCACAT	21
CD28	CD28 F	TCAAAGGAGTTCCGGGCATC	20
	CD28 R	CTGAAGCAGGCGGGAGTAAT	20
CD19	CD19 F	GCAGGTCCTCTTCCAGTCAC	20
	CD19 R	TCAGGATCAGGGCTCTTCGA	20
CD21	CD21 F	TGCCATGCCTACAAAGCTGA	20
	CD21 R	GTAGTAACCAGGGCGGCATT	20
CD81	CD81 F	CCAGACTACCAGCCTCCTCT	20
	CD81 R	GCACAGTACCATGCTCAGGA	20
C3d	C3d F	ACAAATTGACCCAGCGTAGG	20
	C3d R	GCACGTCCTTGCTGTACTGA	20
HPRT	HPRT F	CCCAGCGTCGTGATTAGTGA	20
	HPRT R	GCCGTTCAGTCCTGTCCATA	20

Figure S1

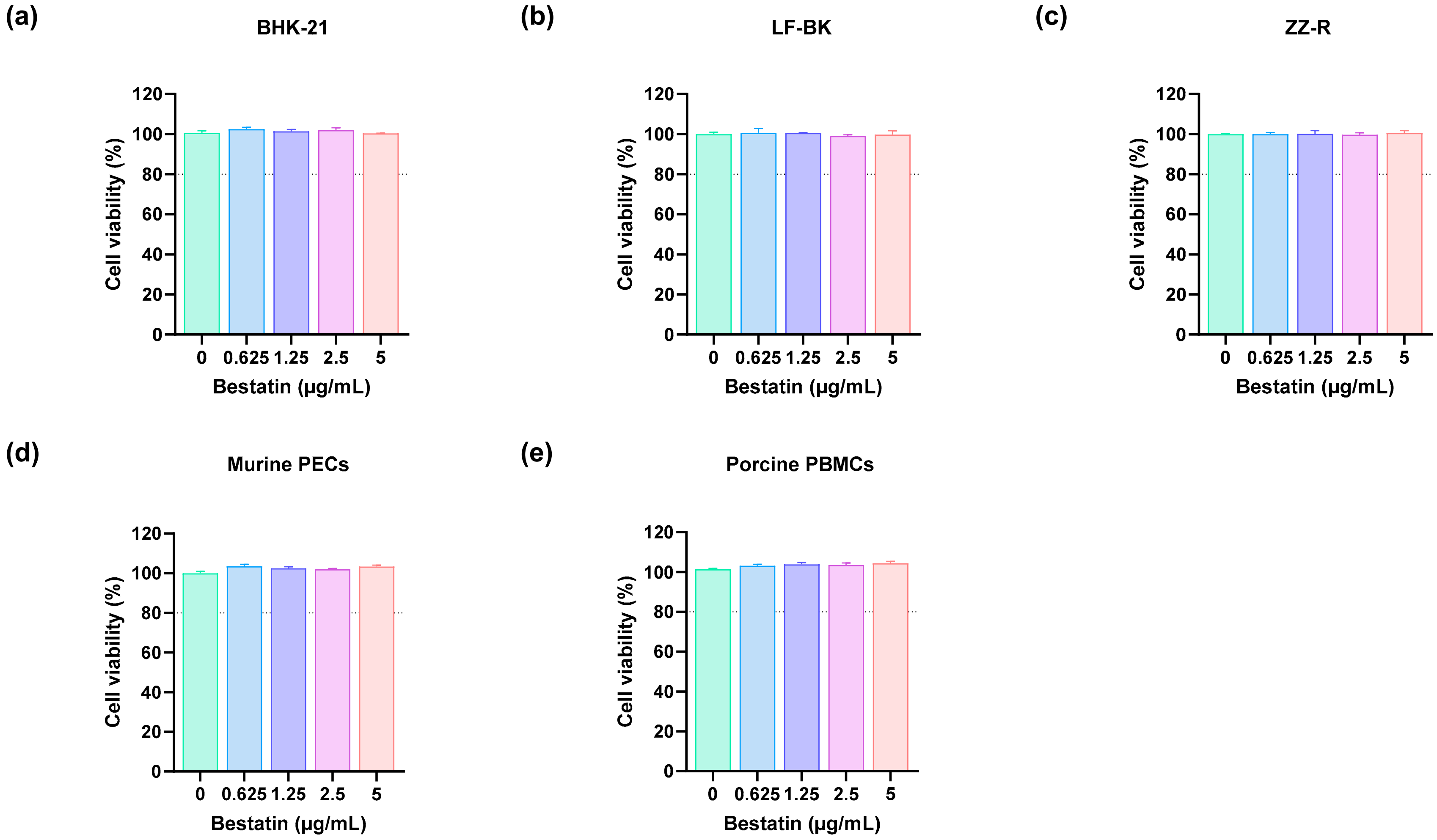

Figure S2

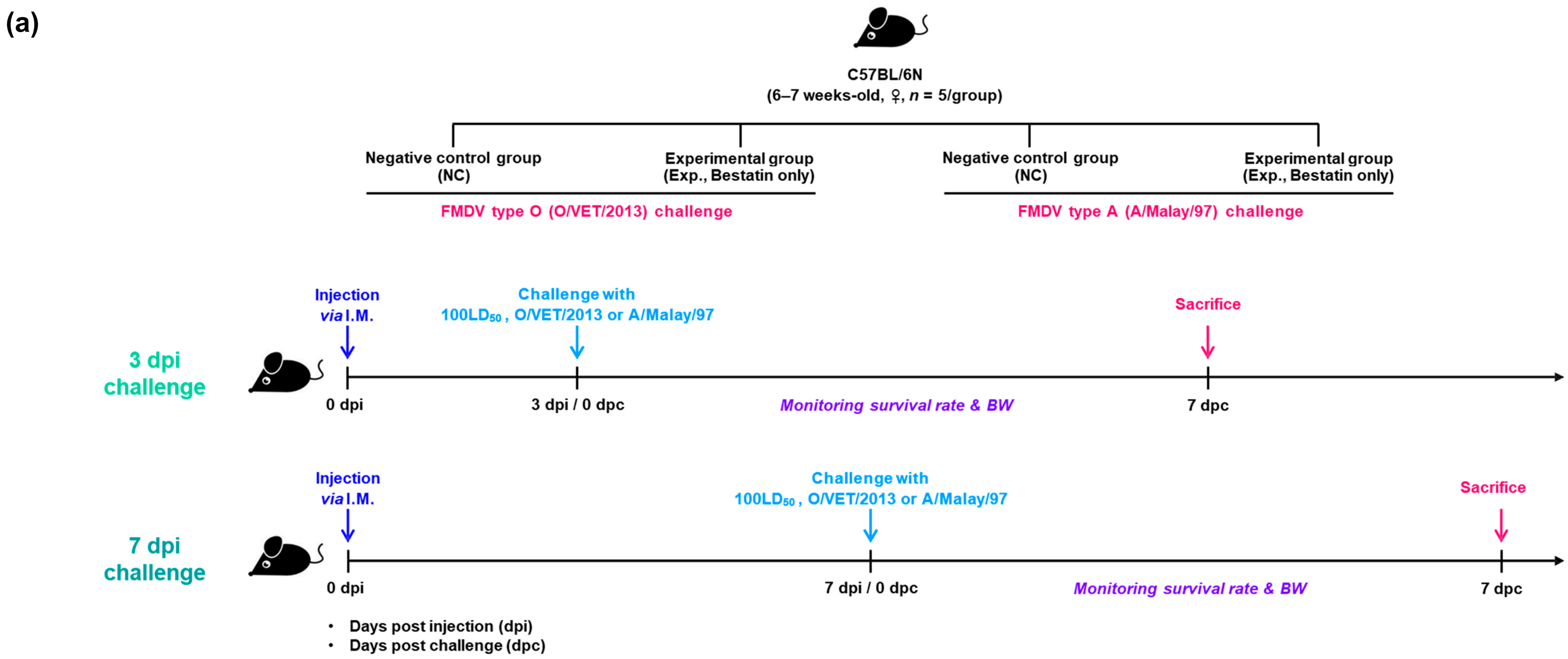
(a)
C57BL/6N
(6–7 weeks-old, ♀, n = 5/group)
Negative control group (NC)
Experimental group (Exp., Bestatin only)
FMDV type O (O/VET/2013) challenge
Negative control group (NC)
Experimental group (Exp., Bestatin only)
FMDV type A (A/Malay/97) challenge
3 dpi challenge
Injection via I.M.
Challenge with 100LD50 , O/VET/2013 or A/Malay/97
Sacrifice
0 dpi
3 dpi / 0 dpc
Monitoring survival rate & BW
7 dpc
7 dpi challenge
Injection via I.M.
Challenge with 100LD50 , O/VET/2013 or A/Malay/97
Sacrifice
0 dpi
7 dpi / 0 dpc
Monitoring survival rate & BW
7 dpc
• Days post injection (dpi)
• Days post challenge (dpc)

Figure S2 (continued)

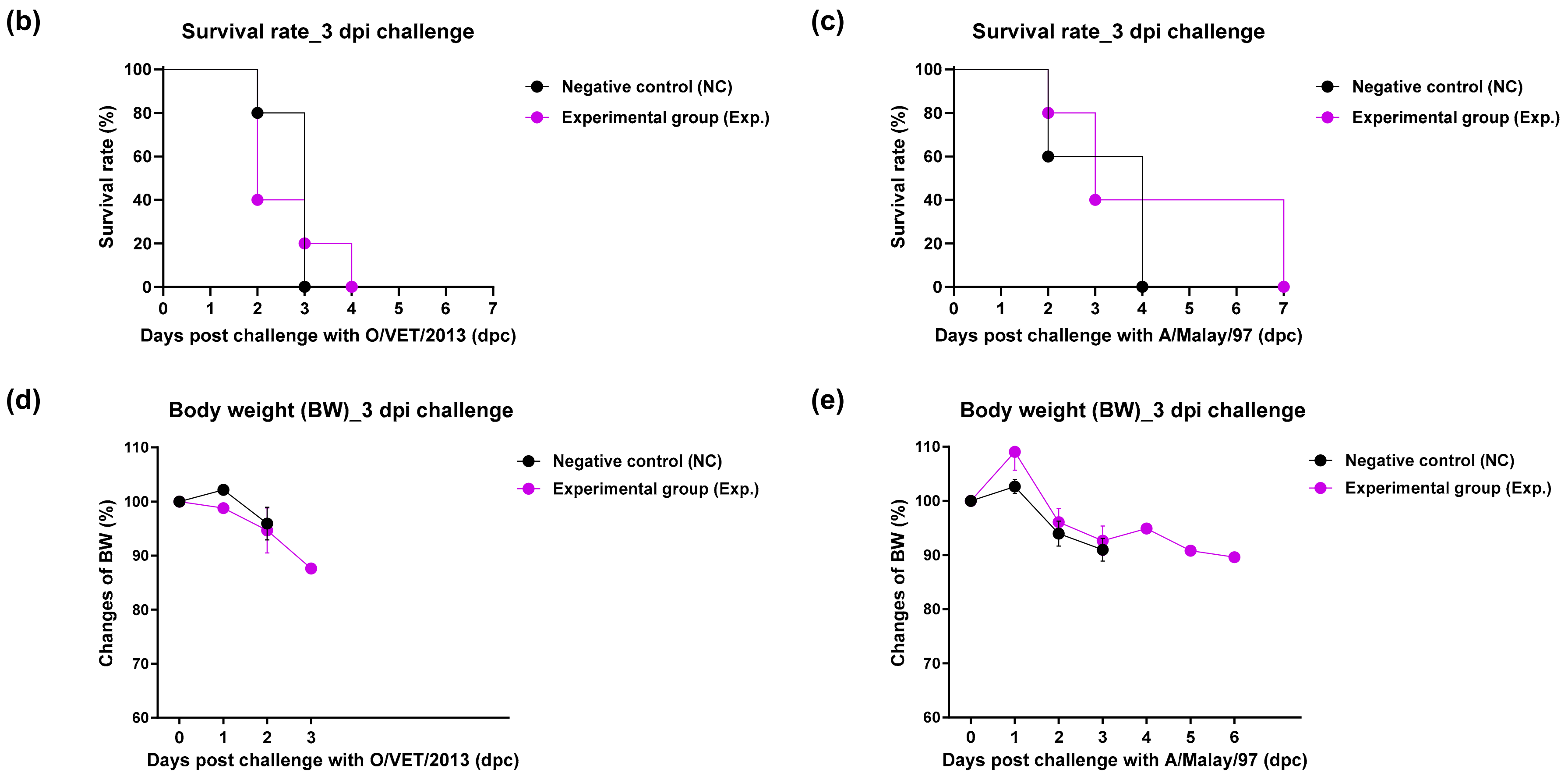

Figure S2 (continued)

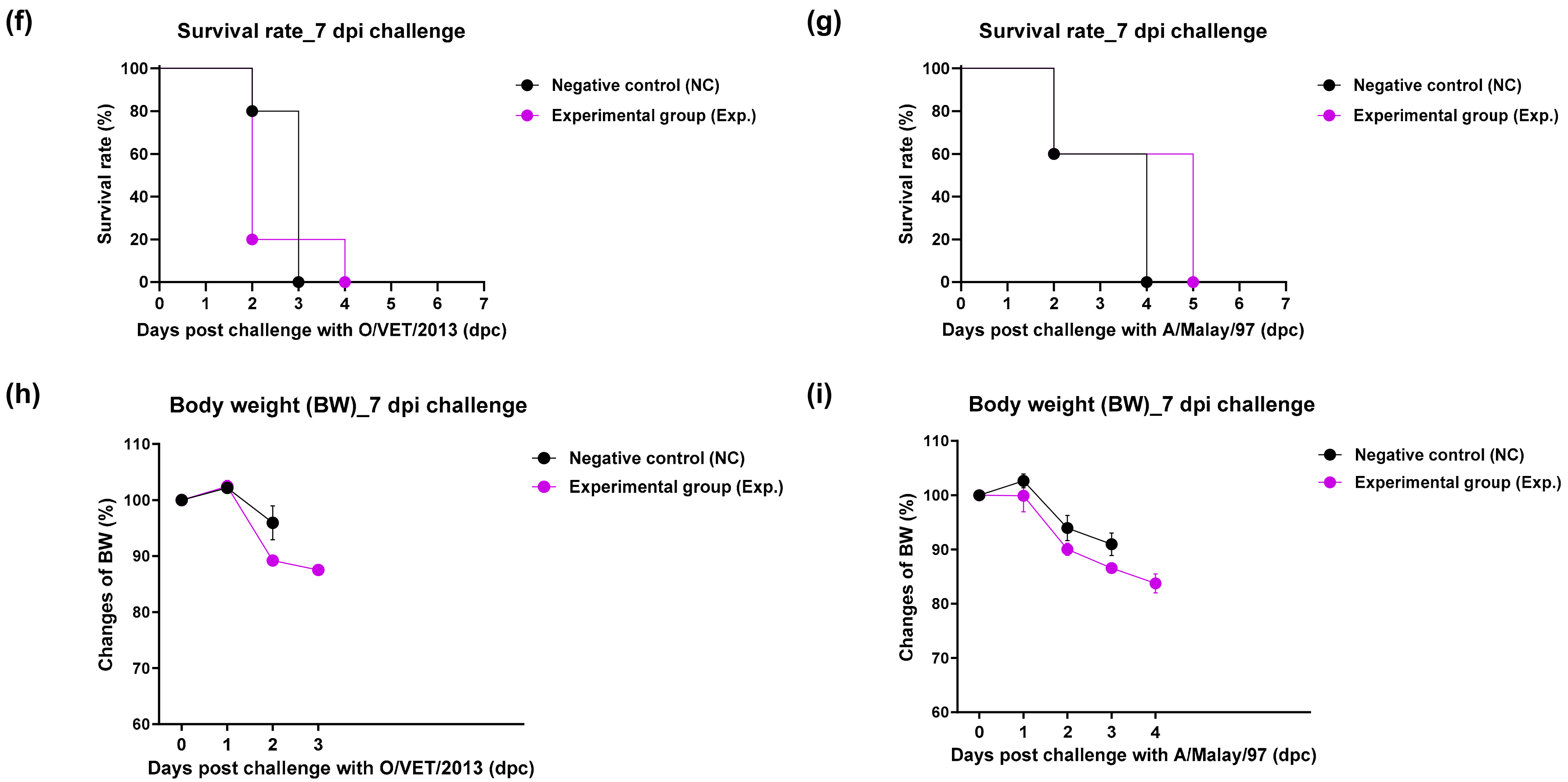

Supplement: Supplementary file 1 [file vaccines-11-01690-s001.zip › vaccines-2644514-supplementary.pdf]
